# Short‐ and long‐term outcomes of polyethylene band attenuation of congenital extrahepatic portosystemic shunts in dogs: 60 cases (2010‐2020)

**DOI:** 10.1111/jsap.13552

**Published:** 2022-09-11

**Authors:** O. Glenn, A. Tomlinson, G. Pinchbeck, R. Burrow

**Affiliations:** ^1^ Royal (Dick) School of Veterinary Studies The University of Edinburgh Easter Bush Campus Midlothian EH25 9RG UK; ^2^ Small Animal Teaching Hospital University of Liverpool, Leahurst Neston CH64 7TE UK; ^3^ Institute of Infection, Veterinary and Ecological Sciences University of Liverpool. Leahurst Neston CH64 7TE UK; ^4^ Northwest Veterinary Specialists Cheshire WA7 3FW UK

## Abstract

**Objectives:**

To report the short‐ and long‐term outcomes following attenuation of congenital extrahepatic portosystemic shunts in dogs using a novel polyethylene band.

**Materials and Methods:**

Records were retrospectively reviewed for dogs that underwent congenital extrahepatic portosystemic shunt attenuation by a polyethylene banding technique, at a single institution between 2010 and 2020. Short‐term outcome data were collected from peri‐operative clinical records with follow‐up examinations, scheduled at 6 and 18 weeks post‐operatively, and post‐operative imaging when performed. Long‐term follow‐up was collected by validated owner questionnaire, telephone interview or medical records. Long‐term outcomes were categorised by “excellent”, “good” or “poor”.

**Results:**

Sixty dogs were included. Post‐operative complications occurred in 10 of 60 dogs (16.7%), four major and six minor, with a peri‐operative mortality of 6.7%. Persistent shunting was identified in nine of 53 dogs (17%) available for follow‐up examination and four dogs underwent a revision surgery. Long‐term follow‐up was available for 44 dogs at a median of 75 months post‐operatively (range 7 to 128). Long‐term outcomes were “excellent” (26) or “good” (8) in 81.8% of dogs and “poor” (8) in 18.2%. At the time of follow‐up, 30 of 44 (68.2%) dogs were not receiving any medical treatment and 27 of 28 (96.4%) questionnaire respondents were satisfied with the response to surgery.

**Clinical Significance:**

Polyethylene band attenuation of congenital extrahepatic portosystemic shunts provides comparable outcomes to cellophane. The material used in this study is widely available and consistent while being pre‐sterilised and pre‐folded makes it easy to use.

## INTRODUCTION

A congenital extrahepatic portosystemic shunt (CEHPSS) is an anomalous vessel that connects the portal venous system directly to the systemic venous system, allowing blood to bypass the liver with resultant clinical signs of liver dysfunction.

Surgical management is preferred to medical management of portosystemic shunts due to improved survival and reduced frequency of clinical signs (Greenhalgh *et al*. 2014). Several surgical options have been described including complete ligation (Bristow *et al*. 2017) as well as gradual attenuation by thin film band (TFB) (Harari *et al*. 1990) or ameroid constrictor (AC) (Vogt *et al*. 1996). As 48% to 82% of dogs cannot tolerate complete ligation at the time of surgery (Hottinger *et al*. 1995, Wolschrijn *et al*. 2000), techniques that achieve gradual attenuation are preferred.

Thin film banding was first reported to treat CEHPSSs in 1990 (Harari *et al*. 1990). It has been shown to gradually occlude vessels through a chronic, low grade foreign body reaction at a slower rate than ACs (Youmans & Hunt 1999), which is desirable to prevent portal hypertension or multiple acquired shunts.

Cellophane was originally reported for use as the TFB but as no uniform source was available, crafting materials were often used (Harari *et al*. 1990, Youmans & Hunt 1999, Smith *et al*. 2013, Nelson & Nelson 2016, Traverson *et al*. 2018, Otomo *et al*. 2020). Smith *et al*. (2013) and Field *et al*. (2019) found that only 15% to 25% of TFBs were actually cellophane. Other materials they identified included polyolefin, polyester, polypropylene, modified cellophane and two unidentified materials. Some studies of thin film banding of CEHPSS did not describe their source of cellophane (Hunt *et al*. 2004, Frankel *et al*. 2006) and many did not analyse the composition (Harari *et al*. 1990, Youmans & Hunt 1998, Hunt *et al*. 2004, Frankel *et al*. 2006, Landon *et al*. 2008, Schaub *et al*. 2016). In addition, as the materials used for TFBs are generally not sourced sterile, they require sterilisation which can cause major alterations in the mechanical properties; depending on the sterilisation technique and composition of the TFB (Smith *et al*. 2013). Reports of TFBs have shown varied clinical results (Landon *et al*. 2008, Nelson & Nelson 2016, Traverson *et al*. 2018), which may be due to many factors such as surgical technique, surgeon experience, TBF material and method of outcome assessment. Only two reports have evaluated non‐cellulose material for thin film banding (polyolefin), both reported good long‐term outcomes (Nelson & Nelson 2016, Otomo *et al*. 2020). This has lead to the suggestion that synthetic materials could cause a greater inflammatory reaction (Berent & Tobias 2017).

Use of polyethylene was reported in 1948 as a film for achieving gradual attenuation of large vessels in human patients (Yeager & Cowley 1948) and then for gradual occlusion of the portal vein dogs (Stone & Murphy 1949). Subsequently, this polymer has been widely used in surgery as sheets for hernia repair (Yeager & Cowley 1948, Collins 1965), tape for tendon augmentation (Leciejewski *et al*. 2018) and as suture material for complete ligation of CEHPSSs (Bristow *et al*. 2019). The terms “polythene”, “polyester” and “cellophane‐polythene” have been previously used to describe polyethylene (Yeager & Cowley 1948, Tan *et al*. 2003). The following databases [Medline (PubMed), Science direct, Ovid, Web of Science] were searched with the following keywords [attenuation, portosystemic and (thin film OR polyethylene OR polythene OR polyester OR cellophane‐polythene)] on July 2, 2022. No reports of polyethylene band attenuation of portosystemic shunts were found.

The objectives of this study were to determine the short‐ and long‐term outcomes following attenuation of CEHPSS in dogs using a novel, thin film, polyethylene band and to present outcomes by multiple formats previously published for TFBs to aid comparison between studies.

## METHODS AND MATERIALS

### Study design

Medical records of dogs that had undergone surgical treatment of CEHPSS were identified from retrospective review of computer records. Inclusion criteria were dogs with a CEHPSS, treated with partial attenuation by polyethylene band, between January 1, 2010 and January 1, 2020. Exclusion criteria were complete attenuation or previous surgery for a CEHPSS. Ethical approval was obtained from the institution's veterinary research ethics committee, reference number VREC956.

### Data extraction

Data extracted from clinical records included age, sex, breed, bodyweight, presenting clinical signs, haematology, biochemistry, bile acid stimulation test (BAST), fasting ammonia when available, duration of anaesthesia, duration of surgery, use of portal venography, use of additional surgical procedures performed, duration of hospitalisation and early post‐operative complications (within 6 weeks post‐operatively). Early post‐operative complications were classified as major (requiring revision surgery or resulting in death) or minor. Shunting vessels were classified as left gastro‐caval, left gastro‐phrenic, porto‐azygos, caudal mesenteric‐caval or unclassified (White & Parry 2013, Berent & Tobias 2017). Data from follow up examinations scheduled at 6 and 18 weeks post‐operatively included clinical signs, medications, haematology, biochemistry and BAST. When BAST was suggestive of persistent shunting, repeat imaging was offered to owners. These data were recorded when available. For dogs requiring a revision surgery following polyethylene band attenuation; the time since first surgery, imaging findings, surgical findings and subsequent method of attenuation were recorded. Revision surgeries were excluded from statistical analysis.

### Surgical procedure

**FIG 1:**
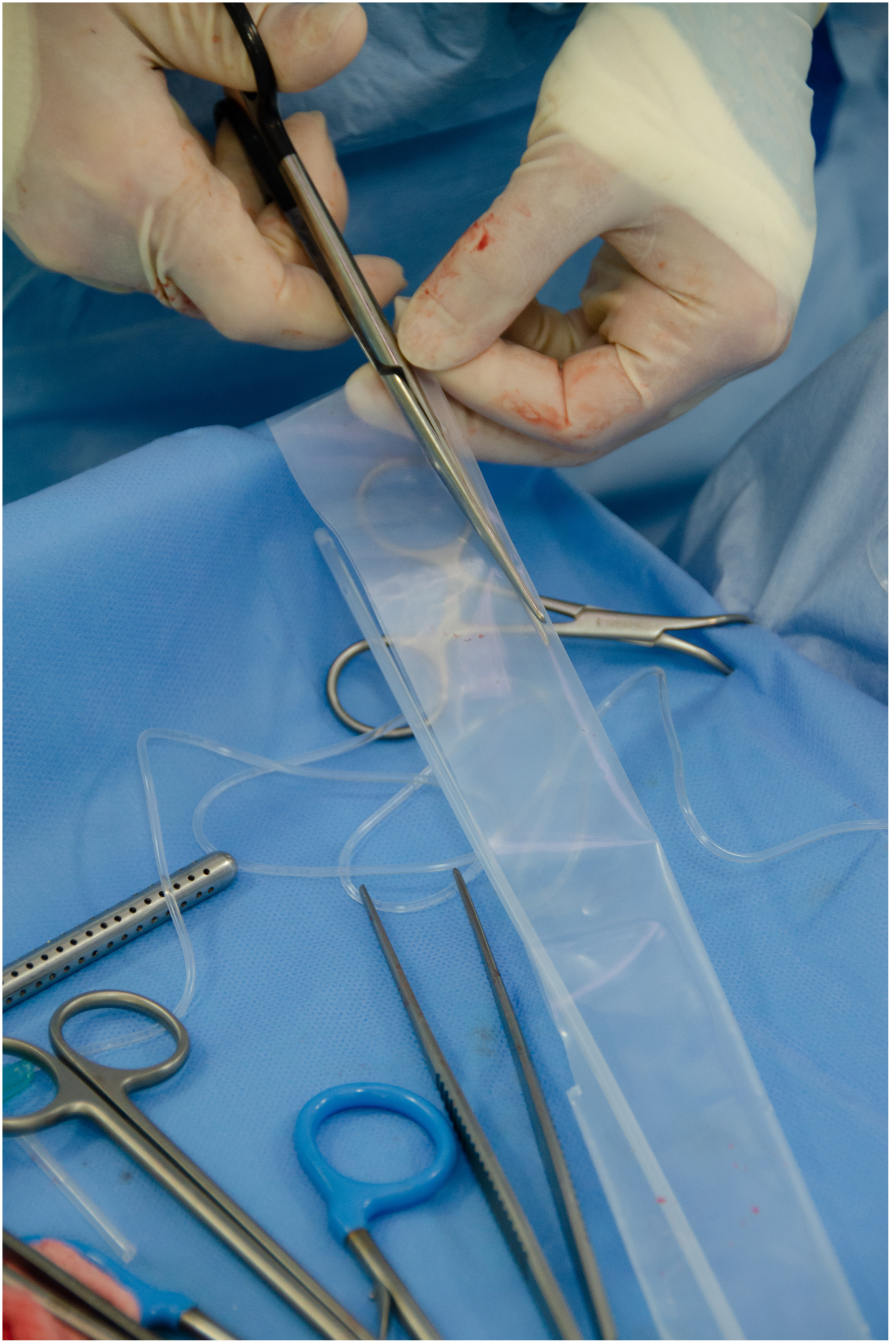
Three‐ to five‐millimetre diameter polyethylene thin film band being cut along pre‐folded edge before placement around a CEHPSS

**FIG 2:**
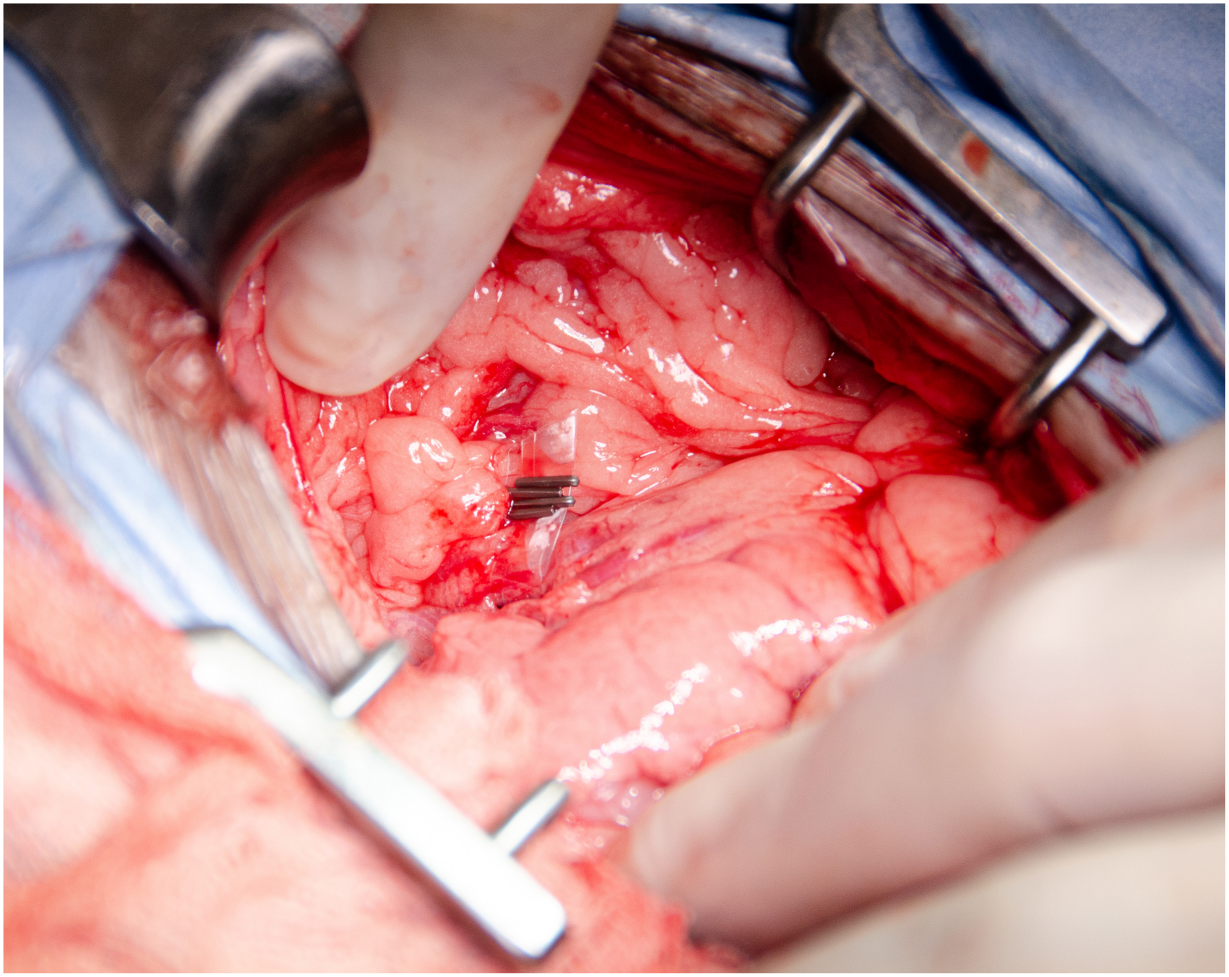
Polyethylene band in place around a CEHPSS and secured with haemostatic clips

All procedures were carried out by a single European College of Veterinary Surgeons specialist in small animal surgery or surgical residents under their supervision, at a single institution. Anaesthesia protocol was at the discretion of the attending anaesthetist. A routine midline celiotomy was performed and the CEHPSS identified visually or with intra‐operative mesenteric portal venography. Right angled vascular forceps were used to dissect around the shunt and a double strand of 3‐metric silk (Mersilk; Ethicon) placed around the shunt and used as a tourniquet. Portal hypertension was assessed for during complete occlusion of the shunt through visual assessment of the pancreas and small intestines and observation of changes in anaesthetic variables as previously described (Youmans & Hunt 1998). Patients who could tolerate temporary complete occlusion without signs of portal hypertension had complete attenuation with 3‐metric silk. In dogs that could not tolerate complete occlusion, a polyethylene TFB was placed. The polyethylene used was the inner sterile packaging material of a 10 French male dog urinary catheter with female luer mount (Portex; Smiths Medical Inc.). A 3 to 5 mm wide strip of polyethylene was cut along a pre‐made folded edge (Fig 1) to create a two‐layered TFB. The TBF was passed around the shunt with right angled vascular forceps and secured in place with three or four haemostatic clips (Ligaclip Extra LT400; Ethicon) (Fig 2). In dogs where some shunt occlusion was tolerated the band was placed to cause partial occlusion of the shunt without inducing visual signs of portal hypertension. Additional surgical procedures were performed when indicated.

Dogs were hospitalised in an intensive care unit for at least 2 days post‐operatively. Vital parameters and bodyweight were monitored in all dogs with additional monitoring (blood glucose, albumin, total protein and abdominal ultrasound) in those with clinical findings suggestive of development of portal hypertension or post‐attenuation neurological signs (PANS). Fasting ammonia was not routinely measured in dogs with PANS.

When death or euthanasia occurred, date and cause of death were collected from clinical records or referring veterinarians to categorise if the event was related to the CEHPSS surgery. Where insufficient information was available, it was recorded as unknown.

### Long‐term follow‐up

Long‐term follow up was sought for dogs alive at least 6 months post‐operatively. Referring veterinarians were asked to confirm if dogs were alive before owner contact. Owners of dogs not confirmed alive were not contacted for ethical reasons and were considered lost to follow‐up at the time of last veterinary contact. Owners were sent an information letter and asked to complete a validated owner questionnaire (Bristow *et al*. 2019) online. If not completed, telephone interviews were conducted with owners 30 days after receipt of the information letter. Where an owner completed a questionnaire after telephone interview, the questionnaire was used. If no owner follow‐up was available after receipt of the information letter and telephone call, medical records from referring veterinarians were reviewed.

Outcomes were categorised as “excellent”, “good” or “poor” as described by Mehl *et al*. (2005). “Excellent” was a clinically normal dog receiving no medical management of hepatic encephalopathy (low‐protein diet, lactulose, antimicrobials). “Good” was a clinically normal dog receiving medical management of hepatic encephalopathy. “Poor” was any dog with clinical signs attributable to a CEHPSS or that died or was euthanased because of their CEHPSS.

Results from the validated owner questionnaire were used to calculate a pre‐ and post‐operative congenital portosystemic shunt (CPSS) score and quality of life (QoL) score as previously described (Bristow *et al*. 2019). The frequencies of clinical signs were recorded on a 5‐point scale of never (0), less than once per month (1), monthly (2), weekly (3) or daily (4). Frequency was then weighted by severity of clinical sign as follows: seizures multiplied by 3; head pressing, ataxia, disorientation, aggression, collapse, unresponsiveness, apparent blindness, fatigue/weakness and circling multiplied by 2; vomiting, diarrhoea, decreased appetite, haematuria and dysuria multiplied by 1. The presence of cystoliths or urethral obstruction had 2 points added. Retarded growth had four points added if present and two points added if owner was unsure. This produced a cumulative CPSS score out of 110, where a higher score represents a more severely affected animal. Owners were then asked to rate their dog's QoL on a visual analogue scale from “worst imaginable” to “best imaginable”; giving an owner perceived QoL score out of 100, where a higher score represents a better QoL.

### Thin film band analysis

A sample of the TFB used in the study and presumed to be polyethylene was analysed using attenuated total reflectance Fourier transform infrared spectroscopy (ATR‐FTIR) with a Vertex 70 FTIR spectrometer with a diamond crystal ATR and OPUS software.

### Data analysis

Continuous data were assessed for normality graphically and descriptive statistics were performed with SPSS Statistics 24.0.0 (IBM, Woking, UK). Normally distributed data are presented as mean with standard deviation and non‐normally distributed data as median with range. Further statistical analysis was not performed due to the low case numbers involved.

## RESULTS

### Study population

Sixty dogs were included with a median age at time of surgery of 12 months (range 11 weeks to 69 months). Sex distribution was 21 entire females, 15 neutered females, 17 entire males and seven neutered males.

Breed distribution included cross bred (8), shih‐tzu's (8), Yorkshire terriers (7), miniature schnauzers (6), Maltese terriers (5), Jack Russel terriers (5), pugs (4), West Highland white terriers (4), Dandie Dinmont terriers (2) and one of each of bichon frise, Border collie, Cairn terrier, cocker spaniel, Dalmatian, golden retriever, Lhasa apso, Norfolk terrier, papillon, Pomeranian and Shetland sheepdog.

Presenting clinical signs were neurological in 34 dogs (56.7%), including altered mentation, ataxia, circling, head pressing, blindness and seizures; gastrointestinal in 34 (56.7%), including vomiting and diarrhoea; urogenital in 15 (25%), including haematuria, stranguria and obstruction; and polydipsia or polyuria in 15 (25%). Seven of the 34 dogs with neurological signs had pre‐operative seizures and six of the 15 dogs with urogenital signs had cystoliths.

CEHPSS morphologies were left gastro‐caval (38), unclassified (13), porto‐azygos (6), left gastro‐phrenic (2), caudal mesenteric‐caval (1).

### Surgical procedure

Median anaesthesia duration was 118 minutes (range 70 to 180) and median surgery duration was 72 minutes (range 30 to 155). Additional procedures performed at the time of surgery were ovariohysterectomy (6), cystotomy (5), voiding hydropulsion (2) and bilateral inguinal cryptorchid castration (1). Dogs remained in an intensive care unit for a median of 5 days (range 2 to 17) post‐operatively. All received antibiotics, lactulose and a protein restricted diet. Levetiracetam was prescribed to 10 dogs, with and without pre‐operative seizures, based upon the evidence of protective effect against PANS available at the time.

### Early post‐operative complications

Early post‐operative complications occurred in 10 dogs (16.7%). PANS developed in five dogs, of which two had seizures; three developed acute portal hypertension; one formed an incisional seroma and one was re‐admitted for vomiting. Four of these complications were major and resulted in death; one dog with PANS died 3 days post‐operatively from respiratory depression, one dog with PANS had global brain ischemia diagnosed on MRI and died 4 days post‐operatively, one dog with PANS and seizures was euthanased 11 days post‐operatively due to uncontrolled seizure activity and one dog that developed acute portal hypertension 4 days post‐operatively was euthanased 47 days post‐operatively due to lack of response to medical treatment and financial constraints. This gave a peri‐operative mortality of 6.7% within 6 weeks of surgery.

### Short‐term follow‐up

**FIG 3:**
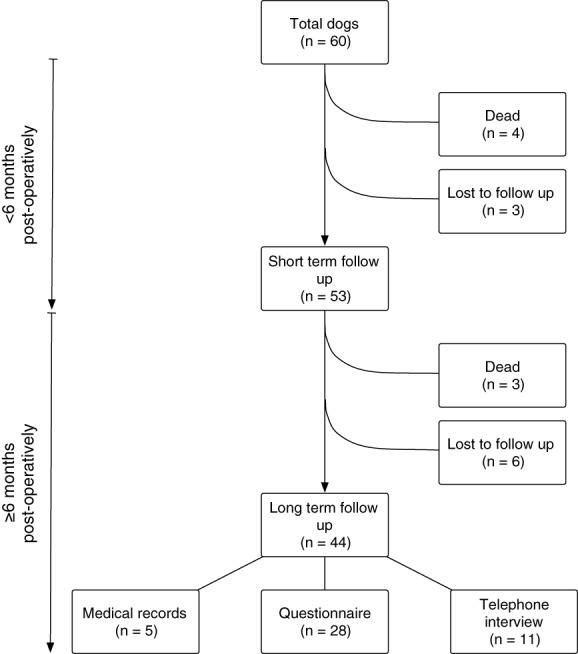
Flow diagram illustrating the number of dogs at each data collection point

Short‐term follow‐up was available for 53 of the 56 remaining live dogs. The number of dogs at each follow‐up point are presented in Fig 3. BAST results are present in Table 1.

**Table 1:** Bile acid stimulation test results of the 53 dogs with short‐term follow‐up

		Pre‐operatively	First follow‐up	Second follow‐up
Time post‐operatively (median with range)		–	7 weeks (3 to 25)	19 weeks (9 to 64)
Number of dogs with available SBA results		57	53	28
SBA (median with range)	Pre‐BAST	119 μmol/L (1.8 to 1588)	15 μmol/L (0 to 472)	15.4 μmol/L (0 to 553)
	Post‐BAST	165 μmol/L (1.7 to 983)	58 μmol/L (1.8 to 743)	59.5 μmol/L (6.7 to 900)
SBA change from pre‐operatively (median with range)	Pre‐BAST	–	−105 μmol/L (−1494 to 195)	−85.6 μmol/L (−713 to 296)
	Post‐BAST	–	−113 μmol/L (−876 to 526)	−83.3 μmol/L (−938 to 562)
SBA % change from pre‐operatively (median with range)	Pre‐BAST	–	−88% (−99.5 to 900)	−84% (−99.7 to 115)
	Post‐BAST	–	−66.3% (−99 to 243)	−53.1% (−95.7 to 255)
% of dogs with SBA reduction from pre‐operatively	Pre‐BAST	–	88.6% (39/44)	96% (24/25)
	Post‐BAST	–	87.5% (35/40)	85% (17/20)
% of dogs with SBA normalisation (<15 μmol/L)	Pre‐BAST	–	59.2% (29/49)	50% (14/28)
	Post‐BAST	–	21.2% (11/52)	14.8% (4/27)

SBA Serum bile acids, BAST Bile acid stimulation test

### Persistent shunting

Persistent shunting was identified in nine of the 53 (17%) dogs available at short‐term follow‐up, at a median of 180 days (range 85 to 1460) post‐operatively. Four dogs were diagnosed by ultrasound scan, three by CT, one by both ultrasound and CT and one by exploratory celiotomy. The causes of persistent shunting were previously unidentified branches or second shunts in three dogs, persistent flow through the attenuated vessel in two dogs, unable to be determined by ultrasound scan in two dogs and unrecorded in two dogs. None had multiple acquired shunts. A revision surgery was performed in four dogs of which two had complete ligation with silk, one had a second polyethylene band placed on a previously unidentified branch and one did not have the method recorded. Long‐term follow‐up was available for three of these four dogs and all were classified as “excellent”.

### Long‐term follow‐up

Forty‐four of the 50 dogs alive at the time of long‐term follow‐up were available, at a median of 75 months post‐operatively (range 7 to 128) and median age of 100 months (range 10 to 154). Median survival time could not be estimated due to the small number of deaths. Three dogs were documented to have died or been euthanased and nine were lost to follow‐up. Of these three dogs, one was euthanased in relation to a mammary mass, one in relation to an oral mass and one died of unknown causes. The number of dogs followed up by questionnaire, telephone interview or medical records are presented in Fig 3.

Outcome classifications are presented in Table 2. At the time of follow‐up, 30 of 44 (68.2%) dogs were not receiving any medical treatment, three were receiving only a low‐protein diet and 11 were receiving other medical management of hepatic encephalopathy.

**Table 2:** Long‐term outcomes of 44 dogs, as classified by Mehl *et al*. (2005)

Outcome classification	Number of dogs	Percentage
Excellent	26	59.1%
Good	10	22.7%
Poor	8	18.2%
Total	44	

Questionnaire follow‐up was completed by 28 owners. The results are presented in Tables 3 and 4. In addition, 27 of 28 respondents were satisfied with response to surgery, 24 said their dog had an improved QoL and 27 said their dog had improvement in clinical signs.

**Table 3:** Impact of surgery on clinical signs, quality of life and behaviour of 28 dogs with CEHPSS

	Pre‐operative (VAS median, IQR)	Post‐operative (VAS median, IQR)
CPSS score*	30.5 (13 to 43.5)	3.5 (0 to 7.5)
QoL score	20 (17.5 to 50)	100 (90 to 100)
Willingness to play	55 (30 to 82.5)	85 (60 to 100)
Willingness to interact with owners	65 (50 to 92.5)	100 (80 to 100)
Willingness to exercise	55 (30 to 80)	85 (60 to 100)
Willingness to interact with other dogs	35 (20 to 72.5)	60 (27.5 to 100)

CPSS Congenital portosystemic shunt, QoL Quality of life, VAS Visual analogue scale, IQR Interquartile range

* Lower score is better

**Table 4:** Impact of surgery on owner perceptions of 28 dogs with CEHPSS

	VAS median, IQR
General improvement following surgery	100 (90 to 100)
Improvement in body condition	100 (70 to 100)
Satisfaction with response to surgery	100 (100 to 100)
Worry over dog's condition	20 (0 to 30)

VAS Visual analogue scale, IQR Interquartile range

### Thin film band analysis

ATR‐FTIR analysis of the TBF confirmed that the sample was polyethylene.

## DISCUSSION

Based on the literature search, this is the first report of polyethylene band attenuation of CEHPSS in dogs.

Our peri‐operative mortality of 6.7% compares favourably to recent reports of CEHPSS TFB partial attenuation; with Matiasovic *et al*. (2020) reporting 9.4% mortality with 53 cases of cellophane, and Otomo *et al*. (2020) reporting 11.8% mortality with 85 cases of polyolefin. The largest cohort of dogs with CEHPSS attenuation by TFB was reported by Hunt *et al*. (2004) who reported a lower 3% mortality with 95 dogs and cellophane. The majority of our peri‐operative deaths were from dogs with PANS, with only two of five dogs surviving to discharge. This is similar to the 27.8% survival to 30 days reported by Mullins *et al*. (2020) for TFBs. Further evaluation of PANS cases with measurement of fasting ammonia was not routinely performed due to the lack of association with PANS (Strickland *et al*. 2018). Only two dogs (3.3%) in our population developed post‐attenuation seizures, lower than the range reported for cellophane and other TFBs of 3.8% to 18% (Youmans & Hunt 1998, Traverson *et al*. 2018, Otomo *et al*. 2020).

In total, 96% and 85% of dogs showed a median reduction of 84% and 53.1% in pre‐ and post‐stimulation SBA, respectively, at a median of 19 weeks post‐operatively. Only 59.2% and 21.2% of these pre‐ and post‐stimulation SBA returned to normal. This is comparable to the 53% and 30% of pre‐ and post‐stimulation SBA normalising reported by Matiasovic *et al*. (2020) and the 11.6% of dogs with normalised parameters of liver function reported by Traverson *et al*. (2018), both with cellophane. However, SBA are an unreliable indicator of complete attenuation and persistently raised SBA can be seen despite complete attenuation (Landon *et al*. 2008, Nelson & Nelson 2016, Bristow *et al*. 2017, Vallarino *et al*. 2019, Devriendt *et al*. 2020).

Persistent shunting was found in 17% of dogs in this study and 7.55% had revision surgery. Two dogs had persistent flow through the attenuated vessel, showing incomplete attenuation by the polyethylene band; and three had unidentified branches or second shunts, showing suboptimal placement of the polyethylene band. This is within the published range of 9% to 47% persistent shunting with cellophane band attenuation (Youmans & Hunt 1998, Traverson *et al*. 2018, Matiasovic *et al*. 2020). The persistent shunting due to previously unidentified branches or second shunts in this study could potentially have been avoid with intra‐operative portal venography. The percentage of dogs with persistent shunting reported here should be interpreted with some caution because the lack of routine post‐operative imaging means some dogs with persistent shunting were likely missed, artificially lowering this. This is similar to previous studies (Youmans & Hunt 1998, Nelson & Nelson 2016, Matiasovic *et al*. 2020, Otomo *et al*. 2020) and was due to increased cost and travel requirements of owners, especially in dogs perceived to be clinically well.

At long‐term follow‐up, a high percentage (82%) of dogs were classified as having “excellent” or “good” outcomes with no clinical signs attributable to CEHPSS at long‐term follow‐up, which is comparable to the 62% to 100% reported for cellophane band attenuation (Traverson *et al*. 2018; Matiasovic *et al*. 2020). A different outcome classification system has previously been used with binary “successful” and “unsuccessful” classifications (Mehl *et al*. 2005, Otomo *et al*. 2020), which combines the “good” and “poor” outcomes into “unsuccessful”. Re‐classifying our cases in this way would give 59.1% “successful” and 40.9% “unsuccessful” outcomes which is similar to the 52% “successful” outcomes reported for polyolefin band attenuation (Otomo *et al*. 2020). Our CPSS and QoL scores also closely compare to cellophane (Matiasovic *et al*. 2020) and suture ligation (Bristow *et al*. 2019). These results suggest that polyethylene band attenuation provides similar long‐term outcomes to those reported for cellophane or polyolefin.

Polyethylene causes a local inflammatory reaction which can be augmented with the application of chemicals (Yeager & Cowley 1948). Diacetyl phosphate is commonly used in the manufacture of cellophane and polyethylene and causes a marked fibrotic reaction (Yeager & Cowley 1948, Dahl *et al*. 1960). Chemical analysis of our TFB revealed it to be polyethylene without a diacetyl phosphate coating, concluding that the inflammatory reaction caused in this population was likely due to polyethylene. Communication with the manufacturer confirmed the material has been consistent for the duration of the study period.

Polyethylene, as used in our study, offers several advantages over other TFBs. It comes pre‐sterilised, negating the need for sterilisation in clinic and the associated detrimental effects (Smith *et al*. 2013). It is pre‐folded, making handling and placement easier intra‐operatively; and it comes from a consistent and widely available source. While it is common practice to fold the TFB into three layers (Youmans & Hunt 1998, Hunt *et al*. 2004, Frankel *et al*. 2006, Traverson *et al*. 2018, Otomo *et al*. 2020), our polyethylene TFB was used as two layers because the film was subjectively thicker than cellophane and was already pre‐folded into two layers.

Limitations of this study include its retrospective nature, small sample size, loss to follow‐up and lack of routine post‐operative imaging. Polyethylene bands were not placed with measurement of portal pressures when partially attenuating, rather by subjective assessment of small intestines and pancreas. Measurement of portal pressures could have been used as an additional step to add objectivity to determining the degree of attenuation tolerated and could have affected the degree of ultimate attenuation. Long‐term follow‐up depended upon owner's memory of events and could have been subject to recall bias or amnesia (Schmier & Halpern 2004). Another limitation is the plurality of follow up methods used. The majority of dogs followed up long‐term were with a validated questionnaire (Bristow *et al*. 2019) but the shorter questioning used in telephone interviews could have elicited different responses and affected outcome categorisation. In addition, follow‐up by medical records alone could have missed clinical signs or medication in these five dogs.

In conclusion, polyethylene band attenuation of CEHPSS resulted in “good” to “excellent” long‐term outcomes with low peri‐operative mortality. It is comparable to previous reports of TFBs with the benefit of being pre‐sterilised and pre‐folded from a consistent and widely available source. Further studies are warranted to investigate the optimum material for gradual vessel attenuation and to verify the results in this study.

### Author contributions

**O. Glenn:** Conceptualization (equal); data curation (lead); formal analysis (supporting); investigation (lead); methodology (equal); project administration (lead); resources (lead); software (lead); supervision (lead); validation (lead); writing – original draft (lead); writing – review and editing (lead). **A. Tomlinson:** Conceptualization (equal); formal analysis (supporting); methodology (equal); project administration (supporting); supervision (equal); validation (supporting); writing – original draft (supporting); writing – review and editing (equal). **G. Pinchbeck:** Data curation (equal); formal analysis (lead); software (equal); validation (equal); writing – review and editing (equal). **R. Burrow:** Conceptualization (lead); methodology (equal); project administration (supporting); supervision (equal); validation (equal); writing – review and editing (equal).

### Conflict of interest

None of the authors of this article has a financial or personal relationship with other people or organisations that could inappropriately influence or bias the content of the paper.
